# Analyzing the Impact of Soft, Stimulating and Depressing Songs on Attention Among Undergraduate Students: A Cross-Sectional Pilot Study in Bangladesh

**DOI:** 10.3389/fpsyg.2019.00161

**Published:** 2019-02-05

**Authors:** Mst. Marium Begum, Md. Sahab Uddin, Jannatul Ferdaush Rithy, Janisa Kabir, Devesh Tewari, Azharul Islam, Ghulam Md. Ashraf

**Affiliations:** ^1^Department of Pharmacy, East West University, Dhaka, Bangladesh; ^2^Department of Pharmacy, Southeast University, Dhaka, Bangladesh; ^3^Department of Pharmaceutical Sciences, Faculty of Technology, Kumaun University, Uttarakhand, India; ^4^Department of Pharmacy, Dhaka International University, Dhaka, Bangladesh; ^5^King Fahd Medical Research Center, King Abdulaziz University, Jeddah, Saudi Arabia

**Keywords:** attention, soft song, stimulating song, depressing song, Numeral Finding test, Typo Revealing test

## Abstract

Music is strongly linked to attention and giving attention can boost intelligence. The purpose of this study was to scrutinize the impact of soft, stimulating, and depressing songs on the attention of students. The study was performed on 280 undergraduate students. Students were divided into 4 groups (i.e., control, soft, stimulating, and depressing) and subjected to 3 songs, soft (That’s My Name), stimulating (Rain Over Me) and depressing (Broken Angel) songs. The Uddin’s Numeral Finding (NF) and Typo Revealing (TR) tests were used to analyze the attention of the students. In the NF, 75.54% attention was exerted by students subjected to stimulating song followed by soft song’s group (i.e., 74.32%) with respect to control group. Amid all groups, the lowest percentage, 70.77% of attention was reported for students subjected to the depressing song. For TR test, stimulating song’s group exerted highest, 45.97% attention, soft song’s group exerted 45.27%, control group exerted 42.70%, and lowest (i.e., 41.54%) attention was exerted by depressing song’s group. In NF test, concerning sex, amid male and female, male exerted higher (77.04%) attention than female but for TR test female exerted higher (i.e., 48.15%) attention for students subjected to stimulating song. Regarding the age of the study in case of NF test for stimulating song’s group, 18–20 years age students exerted highest, 82.07% attention but for TR test highest, 48.75% attention was reported for 23–25 years age students. For NF test, regarding the age of the study 1st-year student exerted highest, 92.44% attention but for TR test highest, 57.33% attention was reported for 3rd-year students for stimulating song’s group. Concerning residential status in both NF and TR tests, for students lived with family subjected to stimulating song exerted highest, 77.93% and 48.6% attention, respectively with respect to students lived without family and remaining groups. This study suggested that song influences the neuronal circuits linked to alert and cognitive functions and the stimulating song has the acme power of increasing attention while depressing song reduces the attention. Therefore, the exciting song can be an operative intervention for enhancing attention, cognitive functions, and treatment of associated neuropsychological disorders.

## Introduction

Attention is one of the most important and complicated cognitive functions that play an essential role in human behavior (Anderson, 2010). It acts as a selection process for all the external and internal events in surroundings which need to be balanced a specific level of consciousness (Kiyonaga and Egner, 2014). Starting to find a certain thing, memorizing and processing information attention plays a vital role (Quak et al., 2015). Attention is usually a passive process (Peper, 1979) and is also very important to put the focus on or divide the focus among diverse activities. There are factors that can change the aspects of attention such as drugs, alcohols, fatigue, and stress. Aging is also a contributing factor in impaired cognitive functions along with decreased attention capacities (Harada et al., 2013; Uddin and Amran, 2018).

Music plays an integral part of life as most people use it to express their deepest emotions. It gives us pleasure, relaxation and helps us in relieving stress and improves the mood and movement to expand health outcomes (Murrock and Higgins, 2009; Swaminathan and Schellenberg, 2015). There are some studies that have identified that teenager’s use popular music to deal with loneliness and to take control of their emotional status or mood (Fuld et al., 2009). Different age groups are interested to listen to different kinds of music which mainly depend on the demand of that age group, time and choice (Roberts and Christenson, 2001).

Previous studies have experimented on the relationship between music therapy and attention where music has been used as the source to reduce difficulties associated to attention like attention deficit hyperactivity disorder, as well as neurorestoration of psychological and neurological disorders (Jackson, 2003; Nizamie and Tikka, 2014; Bringas et al., 2015). Further, the inadequate quantity or complete absence of studies inspecting the effects of music on attention leads to several methodological limitations which may account for the extensive inconsistencies in the already existing literature. Though it is believed that listening to music has an inherent ability to reduce stress, however, its effect on attention among the professional students has not conducted so far and it will be too premature to make a conclusion on the beneficial or adverse role of any specific kind of music on attention.

Pharmacy is a vital human service profession with an exceptional position in serving healthcare and functionality to patients. Some essential trait like empathy, deep knowledge of drugs, and lifelong learning skills are recognized as the core competency for the pharmacists and should be developed throughout graduate school (Waterfield, 2010; Tamayo et al., 2016). Deep knowledge of drugs, updates on the new drugs and deep learning skills required substantial attention which is very much required for the production of high-quality work. Different music may have different effects on attention; therefore, this cross-sectional pilot study was designed to explore the relationship between soft, stimulating and depressing songs and attention performance on the attentive abilities of the undergraduate pharmacy students of the four universities namely East West University, Southeast University, North South University and Atish Dipankar University of Science and Technology located in the capital of Bangladesh.

To the best of our knowledge, this kind of endeavor has not been attempted thus far. We hypothesized that students of pharmacy who listened to soft, stimulating and depressing songs would exhibit a different attentiveness when compared to control groups, i.e., without any songs. We also studied the effect of different music on the students based on various variables like age, sex, year of study, and residential status. Here we describe an easy effective model for evaluating the attention among students.

### Theoretical and Conceptual Framework

#### Numeral Finding Test

The NF test represents how subjects tend to consider and analyze the given numbers. The test was developed by Uddin et al. (2016). This test can identify perceptive and remembering mode of the subject by differentiating desired object figures from the whole content in which they are given. Content field is a very confusing owing to copious object figures with double digits. Among the content field, individuals have to consider each object figures as component parts. The perceptive power of the subject depends on the relative speed of processing, remembering power, and finding capacity. A subject’s attention in this test is determined by finding of the object, elimination of unwanted object and calculation of desired object figures (wanted numerals) within a given time frame (Uddin et al., 2016). A previous study using NF test reported markedly (*P* < 0.01; *P* < 0.001; *P* < 0.01) attention-enhancing capacity of nootropic phytoconstituents in human participants (Uddin et al., 2016). In a study, Alam et al. (2018) found that in the NF test 1st-year undergraduate students with last semester cumulative grade point average of only 3.7 exerted 89.73% attention. On the other hand, undergraduate students with >22 years exerted 70.55% attention in NF test with last semester cumulative grade point average of 3.56 (Alam et al., 2018).

#### Typo Revealing Test

The TR test represents how subjects tend to comprehend the given passage and analyze. The test was developed by Uddin et al. (2016). This test determines the interaction of concentration, learning process, and retention power. The mode of presentation i.e., structure and organization of the contents are imperative because when subject read the contents, their attention might divide into the object figures. For analytic learner with quick processing capacity, the separation of the desired object figures offers an advantage. This test identifies the habitual mode of representation of content in memory, the speed of processing and finding ability. A subject’s attention in this test is determined by memorizing power, retrieval capacity and finding of desired object figures (typological mistakes), within a given time frame (Uddin et al., 2016). In adult humans an earlier study performed by TR test revealed significantly (*P* < 0.01; *P* < 0.001; *P* < 0.01) effective attention-enhancing capacity of cognitive enhancing phytoconstituents (Uddin et al., 2016). In a study of academic performance and attention, both male students exerted 46.6% attention in TR test but the cumulative grade point average of the female students were lower than male (Alam et al., 2018). In the TR test, 50.33% attention was exerted by 1st-year undergraduate students with last semester cumulative grade point average of 3.7 (Alam et al., 2018).

## Materials and Methods

### Study Site and Duration

The study was conducted on undergraduate pharmacy students of East West University, Southeast University, North South University and Atish Dipankar University of Science and Technology located in Dhaka Bangladesh. The period of this study was from March to October 2017.

### Study Population and Experimental Design

Out of total 327, 280 students were interested to participate in this study and among these selected students there were not subjected to any severe ailments within the previous 3 months and were devoid of any neuro/psychological deficits. The participants were selected based on the simple random sampling technique which is an unbiased sampling technique and provides an equal probability to each individual for selection. In this study, based on the preference of the students, three songs were selected as soft, stimulating, and depressing songs. The students were divided into 4 groups with 70 students in each as follows - Group 1 (Control): subjected to NF and TR tests without any songs; Group 2 (Soft): subjected to NF and TR tests using the soft song, That’s My Name (Akcent); Group 3 (Stimulating): subjected to NF and TR tests using the stimulating song, Rain Over Me (Pitbull featuring Marc Anthony); and Group 4 (Depressing): subjected to NF and TR tests using the depressing song, Broken Angel (Arash featuring Helena). The demographic data about student’s sex, age, year of study, residential status, and study associated info were also collected.

### Numeral Finding Test

The NF test was performed according to the method of Uddin et al. (2016) that is based on determining the wanted numerals amid a set of numbers. In this test, 100 numbers with double digits were placed randomly in printed papers and were given to the subject. In these 100 numbers, few numerals (i.e., 14, 50, and 62) were repeated several times called wanted numerals (see Supplementary Materials). The time taken by the students, to determine the wanted numerals was measured as Numeral Finding time (NFT). The extent of this test was 3 min. Gradual abate in NFT indicated the improvement of attention. Arise in the percentage of attention is measured of progressed attention. The following equation was used to calculate the percentage of attention (Uddin et al., 2016):

Attention (%):Iwn×100/Gwn

where, Iwn = The sum of the identified accurate wanted numerals, Gwn = The sum of the given wanted numerals.

### Typo Revealing Test

The TR test was performed according to the method of Uddin et al. (2016) that is based on determining the typing mistake from a passage. In this test, a standard passage containing 250 words was given to the students to read prudently at a glance. Then students were subjected to the same text with typological mistakes (see Supplementary Material). The time taken by the students, to determine the typological mistakes was measured as typo finding the time (TFT). The extent of this test was 3 min. Gradual abate in TFT indicated the improvement of attention. A rise in the percentage of attention is measured of progressed attention. The following equation was used to calculate the percentage of attention (Uddin et al., 2016):

Attention (%):Itm×100/Gtm

where, Itm = The sum of the identified typological mistakes, Gtm = The sum of the given typological mistakes.

### Statistical Analysis

Results were collected and lastly assembled and presented as mean ± SEM. SPSS 15.0 (Chicago, IL, United States) and Microsoft Excel 2010 (Roselle, IL, United States) was used for statistical and graphical evaluations. A Z-test was used to find the magnitude of groups and Chi-square test was performed to find relationships between variables. A probability of *P* < 0.05 was considered as statistically significant compared to the control group. In this experiment, the Chi-square test was used because this is a distribution-free (non-parametric) tool which is designed for the analysis of group differences when the measurement of the dependent variable is at a nominal level. Moreover, the main prior assumption that was not meet the parametric test was that the data was non-normal additionally, due to small sample size we are not confident that the data is normally distributed, thus non-parametric test was found suitable. This test is a robust test with respect to the data distribution more specifically, this test does not require the equality of variances or homoscedasticity in the data which permits the statistical evaluation of multiple group studies and dichotomous independent variables both (McHugh, 2013). In this study, we wanted to test the hypothesis that rate of attention after listening to all type of music i.e., soft, stimulating, and depressive is similar with different selected variables like sex, age, year of study, and residential status among pharmacy students. Here sex, age, year of study, and residential status are four observations that are classified according to two specific characteristics TR and NF tests and for evaluating the independence of two attributes. Chi-square test seems of optimum importance for analyzing the data obtained from the TR and NF test.

## Results

Amid the students of this study maximum, 55.36% were male and the remaining 44.64% were female. The age groups of the 47.86% students were in the age group 23–25 years. Maximum participants, 36.79% were 4th (i.e., final) years students and highest 52.86% lived without family. In Table 1, the demographic profile of the students is listed. In Table 2, the effect of various songs (i.e., stimulating, soft, and depressing) on the attention of the students based on NF test is given. 74.32% attention was reported amid students subjected to the soft song, which is higher than the control group. For students subjected to stimulating song highest (75.54%) attention was reported. Among all groups lowest, 70.77% attention was reported among students subjected to depressing songs.

**Table 1:** The demographic profile of the students (*n* = 280).

Parameter	*n*	%
**Sex**		
Male	155	55.36
Female	125	44.64
**Age (years)**		
18–20	37	13.21
21–22	109	38.93
23–25	134	47.86
**Year of study**		
1st year	47	16.79
2nd year	60	21.43
3rd year	70	25.00
4th year	103	36.79
**Residential status**		
With family	132	47.14
Without family	148	52.86

**Table 2:** The effect of stimulating, soft, and depressing songs on the attention of the students using NF test.

Groups	Sex		Age (years)			Year of study				Residential status		NF test
	Male	Female	18–20	21–22	23–25	1	2	3	4	With family	Without family	% of attention (Avg ± SEM)
Control	41	29	11	30	29	17	14	23	16	34	36	71.61 ± 1.981
Soft	32	38	4	26	40	3	10	17	40	31	39	74.32 ± 1.592^∗^
Stimulating	44	26	13	24	33	10	28	6	26	30	40	75.54 ± 1.691^∗∗∗^
Depressing	38	32	9	29	32	17	8	24	21	37	33	70.77 ± 1.75^∗∗^

*Values were expressed as mean ± SEM. ^∗^P < 0.05, ^∗∗^P < 0.01, ^∗∗∗^P < 0.001 significant difference from the control group.*

**FIGURE 1:**
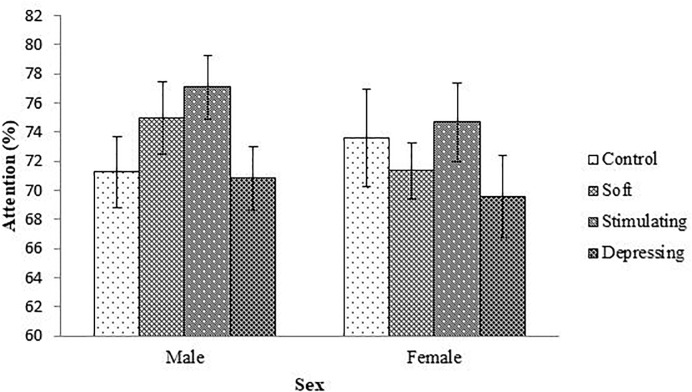
The effect of stimulating, soft, and depressing songs on the attention with respect to the sex of the students using NF test. Values were expressed as mean ± SEM.

**FIGURE 2:**
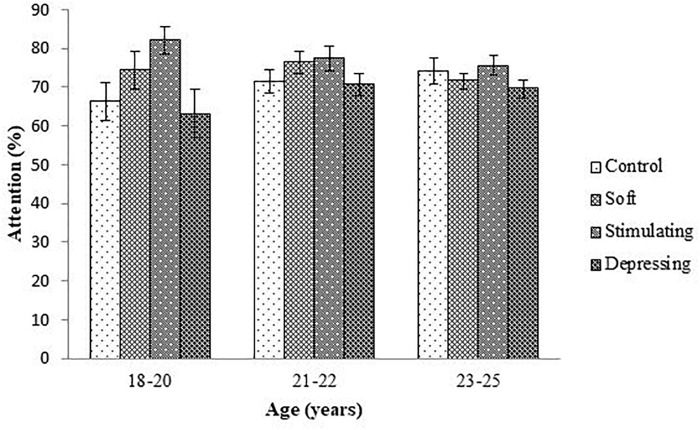
The effect of stimulating, soft, and depressing songs on the attention with respect to the age of the students using NF test. Values were expressed as mean ± SEM.

**FIGURE 3:**
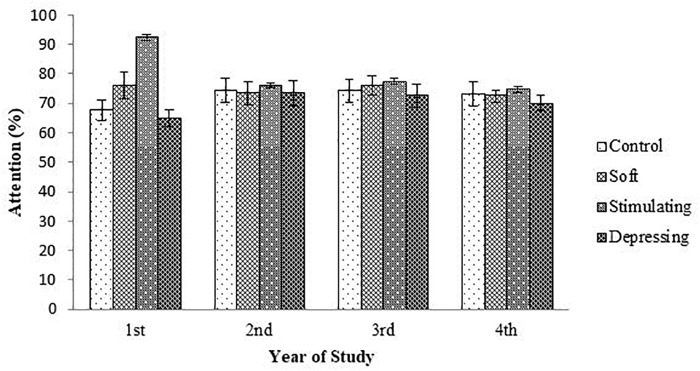
The effect of stimulating, soft, and depressing songs on the attention with respect to the year of study of the students using NF test. Values were expressed as mean ± SEM.

**FIGURE 4:**
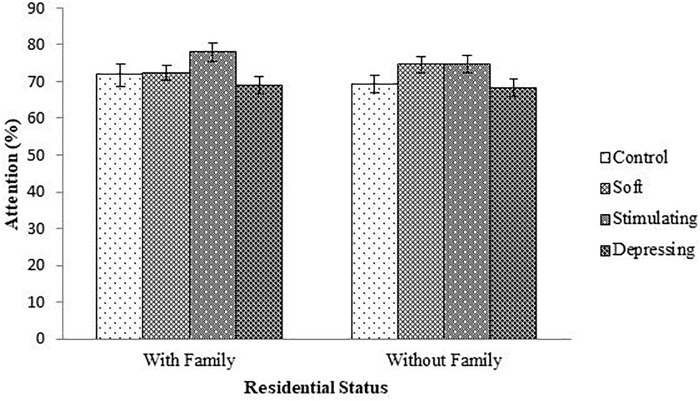
The effect of stimulating, soft, and depressing songs on the attention with respect to the residential status the students using NF test. Values were expressed as mean ± SEM.

The denouements of the song on the attention of the student based on NF test compared to sex, age, year of the study, and residential status are presented in Figure 1–4, respectively. Amid male and female of stimulating song’s group, male exerted highest, 77.04% attention compared to female. Furthermore, the percentage of attention of this group was also higher with respect to remaining groups (Figure 1). In the group subjected to stimulating song, highest percentage (i.e., 82.07%) of attention was reported for 18–20 years aged (Figure 2) students, and the following sequence: 1st (92.44%) > 3rd (77.33%) > 2nd (76.07%) > 4th (74.73%) years students (Figure 3) was reported considering year of study and students lived with family (Figure 4) exerted highest, 77.93% attention. There was no significant association amid sex, age, residential status, and study groups but a highly significant relation was reported for the year of study (Table 3).

**Table 3:** Results of Chi-Square tests concerning sex, age, year of study, and residential status of the students for NF test.

Variable	Value	df	Asymp. Sig. (2-sided)
Sex	4.552	3	0.208
Age	7.613	6	0.268
Year of study	51.905	9	0.000
Residential status	1.720	3	0.633

*df, degrees of freedom; Asymp. Sig., Asymptotic Significance.*

**FIGURE 5:**
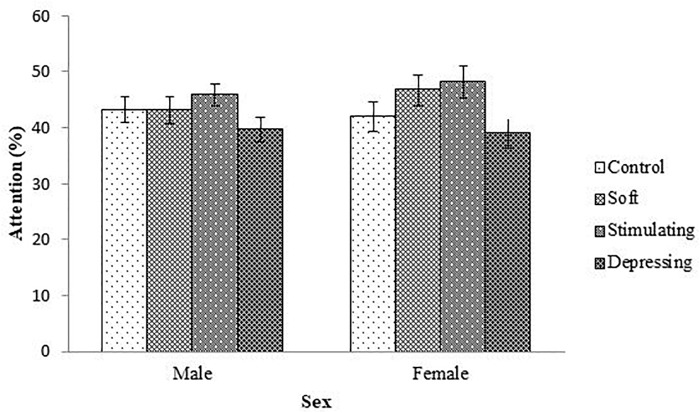
The effect of stimulating, soft, and depressing songs on the attention with respect to the sex of the students using TR test. Values were expressed as mean ± SEM.

**FIGURE 6:**
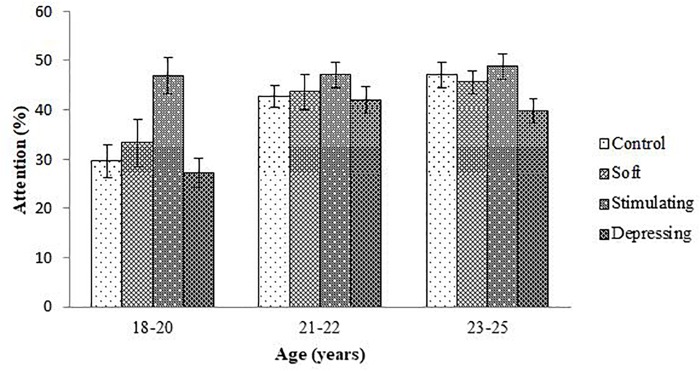
The effect of stimulating, soft, and depressing songs on the attention with respect to the age of the students using TR test. Values were expressed as mean ± SEM.

**FIGURE 7:**
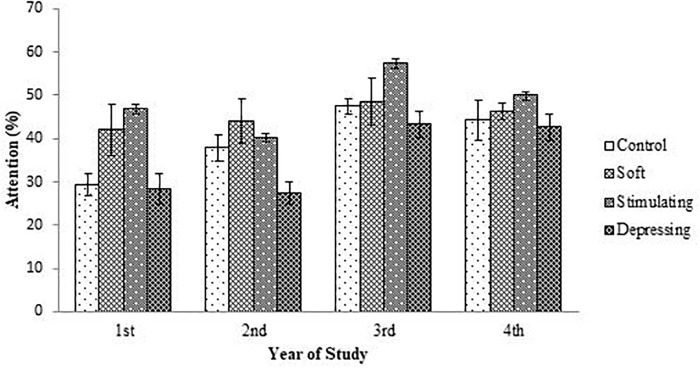
The effect of stimulating, soft, and depressing songs on the attention with respect to the year of study of the students using TR test. Values were expressed as mean ± SEM.

**FIGURE 8:**
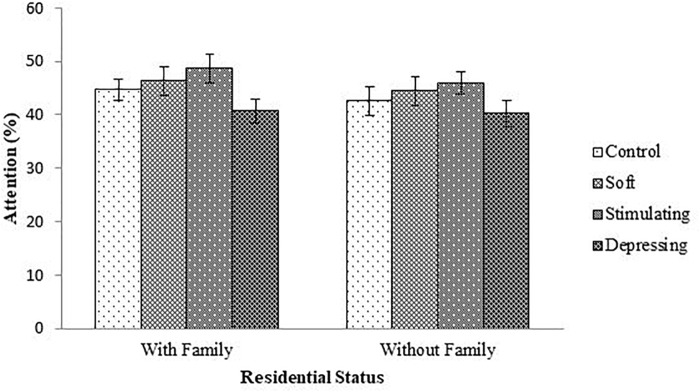
The effect of stimulating, soft, and depressing songs on the attention with respect to the residential status the students using TR test. Values were expressed as mean ± SEM.

The effect of songs on the attention of the student subjected to TR test is given in Table 4. In this test among all groups, lowest, 41.54% attention was reported in student subjected to depression song. However, like the previous test, the highest 45.97% attention was reported among student subjected to stimulating song. The attention of the students subjected to the soft song was 45.27%. Based on TR test, the outcomes of the song on the attention of the student with respect to sex, age, year of the study, and residential status are presented in Figure 5–8, respectively. Regarding sex for stimulating song’s group, the highest percentage of attention (i.e., 48.15%) was reported for female compared to male (Figure 5). Regarding ages for stimulating song’s group, highest, 48.75% attention was reported for 23–25 years aged (Figure 6) students, and considering year of study the following sequence: 3rd (57.33%) > 4th (49.88%) > 1st (46.77%) > 2nd (40.21%) years students (Figure 7) was reported and like NF test, highest, 48.6% attention was exerted by students lived with family (Figure 8). There was no significant association amid sex, residential status and study groups but a highly significant relation were reported for age, and year of study (Table 5).

**Table 4:** The effect of stimulating, soft, and depressing song on the attention of the students using TR test.

Groups	Sex		Age (years)			Year of study				Residential status		TR test
	Male	Female	18–20	21–22	23–25	1	2	3	4	With family	Without family	% of attention (Avg ± SEM)
Control	41	29	11	30	29	17	14	23	16	34	36	42.70 ± 1.69
Soft	32	38	4	26	40	3	10	17	40	31	39	45.27 ± 1.90^∗^
Stimulating	44	26	13	24	33	10	28	6	26	30	40	45.97 ± 1.63^∗∗∗^
Depressing	38	32	9	29	32	17	8	24	21	37	33	41.54 ± 1.68^∗^

*Values were expressed as mean ± SEM.^∗^P < 0.05, ^∗∗∗^P < 0.001 significant difference from the control group.*

**Table 5:** Results of Chi-Square tests concerning sex, age, year of study, and residential status of the students for NF test.

Variable	Value	df	Asymp. Sig. (2-sided)
Sex	3.786	4	0.315
Age	1.257	6	0.000
Year of Study	68.501	6	0.000
Residential Status	2.655	1	0.245

*df, degrees of freedom; Asymp. Sig., Asymptotic Significance.*

## Discussion

The most interesting area of experiments for neuroscientists is the realization and understanding of the relationship among attention, consciousness and brain learning. Attention is considered as the foremost courses of the dynamic brain (Grossberg, 1999). Music is a vital element of daily life. There are tons of benefits that one originates from listening to music (Scarantino, 1987). From pain management to improved sleep quality, listening to classical music possesses both mental and physical benefits. Moreover, creativity, productivity, and mood can be particularly impacted by listening to classical music as contextual sound (Ritter and Ferguson, 2017). In this current study, we used NF and TR tests to scrutinize the relationship between attention and various songs in students.

In this experiment, NF test was used to determine the attention performance that is based on the identification of the wanted numbers repeated numerous times among the 100 numbers. In order to identify as well as calculate each repeating number, more attention is compulsory. This study suggested that in the NF test percentage of attention was higher in stimulating song’s group followed by soft song’s group with respect to control group. Amid all groups, the lowest percentage of attention was reported for depressing song’s group. Listening to memory boosting music during the course of study is linked to getting excessive levels of expectations. Verrusio et al. (2015) examined the impact of Mozart’s music on brain functions by examination of the spectral of electrocardiography amid typical young adults, elderly and elderly with mild cognitive impairment. The researchers reported that listening to Mozart was associated with the rises of alpha band as well as median frequency index of related alpha rhythm action (i.e., a form of brainwave bustle connected to cognition, memory as well as open mind to difficult resolving) was observed both in young adults and elderly. This denouement is probably the representative of the fact that Mozart’s music is capable to stimulate the neuronal circuits linked to attention and cognitive functions (Verrusio et al., 2015).

Males and females are exclusive regarding interpretation. Cognitive function, behaviors and mental functions of male and female are diverse (Uddin, 2017). In this study, for NF test concerning sex, the percentage of attention was higher in stimulating song’s group followed by soft song’s group except for female students with respect to control group and lowest percentage of attention was reported for depressing song’s group. Amid male and female for stimulating song’s group percentage of highest attention was reported for male students. Goldstein et al. (2001) stated that hippocampus which is responsible for the formation of memory is larger in male than female.

Aging is an important factor for attention (Riddle, 2007). The ages of the students range from 18 to 25 years. Like sex, similar denouements (i.e., stimulating > soft > control > depressing) were reported when students were arranged with respect to age of the students except for 23–25 year age group of the soft song’s group. Regarding year of study, 2nd and 4th-year students of soft song’s group exerted percentage of lowest attention compared to control group but for remaining groups percentage of highest attention was reported for stimulating song’s group and depressing song’s group exerted the lowest attention. In the stimulating song’s group amid 1st to 4th-year student’s percentage of highest attention was reported for 1st-year students but for depressing song’s group percentage of attention was lower. Generally, attention increases over time owing to the development of brain function as well as increases of conception up to a certain period of age after that decline (Arain et al., 2013; Posner et al., 2014).

In a study, Thompson et al. (2014) reported that the human brain begins to slow down at age 24 with the decline of cognitive-motor functions. Concerning residential status the students for stimulating song’s group, the percentage of attention was higher in students lived with family compared to students lived without family. In the development of cognitive functions and academic achievement, the family is intensely imperative (Singh, 2011; Donnelly et al., 2016). A significant difference is observed between a teenager and an adult’s choice of music but the purpose remains more or less the same. The diversity of music also comes from various ethnicity, culture, and tradition. Research has indicated that there also is a difference in these variables among the genders (Roberts and Christenson, 2001). Female teenagers are more likely than male adolescents to use music to reflect their emotional state, in particular when feeling lonely or upset (Gibson et al., 2000; Roberts and Christenson, 2001). On the contrary, male teenagers are more likely to use music as a stimulant in order to boost their energy levels (Wells and Hakanen, 1991; Roberts and Christenson, 2001).

The TR test was used in this study for the determent of attention that is based on the identification of typing mistakes from a given text. So as to identify as well as calculate the typos, more attention is compulsory. This study suggested that in the TR test, the percentage of attention was higher in stimulating song’s group then soft song’s group compared to control group and lowest percentage of attention was reported for depressing song’s group. In a study, Riby (2013) examined the Vivaldi’s Four Seasons to detect the magnitude of music contact on cognition linked aptitudes. The denouement reported that amid four concertos (i.e., Spring, Summer, Autumn, and Winter) only Spring, mainly the well-accepted, vivacious, sensitive, and inspiring first movement, had the capacity to improve mental attentiveness as well as memory boosting possessions (Riby, 2013).

In TR test, regarding sex, the percentage of attention was higher in stimulating song’s group and male students of soft song’s group excreted the lowest attention compared to the control group. The percentage of attention increases gradually with the increases in the age of the students for stimulating and soft songs and control groups. Regarding year of study, depressing song’s group exerted the lowest percentage of attention compared to the control group but the highest percentage of attention was reported for stimulating song’s group. Amid 1st to 4th years of stimulating song’s group, 3rd-year students exerted the highest percentage of attention. Typical aging is linked to declining of copious functions comprising sensory, motor, physical as well as the psychological act (de Magalhaes, 2004; Seidler et al., 2010; Murman, 2015). Commodari and Guarnera (2008) reported that aged people especially over 60 years age display progressive reducing in the handling of intricate tasks.

Like NF test, regarding residential status in this test a positive association was reported amid attention and family linkage for stimulating song’s group students lived with family exerted the highest percentage of attention compared to students lived without family. Our short-term memory system is such that it provides us with a buffer allowing us to hold on to information for a longer time period before focusing on to other information (Taylor, 2013; Schon et al., 2015). Execution of two or more tasks simultaneously is known to be as multitasking. According to the study, it showed that people are more inclined to make mistakes during multitasking along with making the work slower than usual (Stoet et al., 2013). It is also suggested in studies that music shows an effective role in influencing the activities of neurotransmitters ultimately influencing the memory and attention capacity (Chanda and Levitin, 2013). From the results of NF and TR tests, we can say that stimulating and soft songs has the power of improving attention and on the other hand depressing song might abate attention.

## Conclusion

Music is the pretty sound that gives us harmony and superior pleasure. The results of this study suggested that stimulating song markedly increased the attention of the students, improved attention was also reported for students subjected to the soft song but lowest attention was reported for students treated by depressing song. The attention-enhancing power of the stimulating and soft songs is probably owing to exciting the brain area linked to attention and enrich the body-mind connection. However, further studies are necessary to detect the exact impact of the song on neuronal circuits and explore the effective therapy for the treatment of attention linked neuropsychological disorders.

Based on the obtained results further research can be conducted to explore the mechanistic insight through estimation of biochemical and neurological changes to evaluate the effect of music on students. Additionally, studies on large population size and different study groups are also warranted with some different songs type. Apart from these categories of stimulating, soft and depressive songs other categories like classical, folk, relaxing, and spiritual music could also be evaluated for their effect in cognitive effects and attention among students with estimation of alterations in the neurotransmitter levels.

## Limitation

The present study was conducted in 4 universities of Bangladesh. It would be best if we could accomplish this study in numerous universities all over the country. A different and larger group of the sample can be used in future studies.

## Ethics Statement

The study protocol was approved by the ethics committee of the Department of Pharmacy, East West University, Dhaka, Bangladesh. The study was conducted in accordance with the ethical standards laid down in the 1964 Declaration of Helsinki.

## Author Contributions

This work was carried out in collaboration between all authors. MU designed the study, wrote the protocol, and prepared the draft of the manuscript. MB, JR, JK, and AI performed the tests, collected the data, and compiled the results. DT revised and improved the manuscript. GA reviewed the scientific contents of the manuscript. All the authors read and approved the final manuscript.

## Conflict of Interest Statement

The authors declare that the research was conducted in the absence of any commercial or financial relationships that could be construed as a potential conflict of interest.
