# Supplementary material for: Analyzing the Impact of Soft, Stimulating and Depressing Songs on Attention Among Undergraduate Students: A Cross-Sectional Pilot Study in Bangladesh
Source: Front Psychol. 2019 Feb 5;10:161. doi: 10.3389/fpsyg.2019.00161 (PMC6371049; doi:10.3389/fpsyg.2019.00161)

**Numeral Finding (NF) Test**

Given figure is the set of 100 numbers including wanted numerals used in the NF test and provided to the students to find wanted numerals.


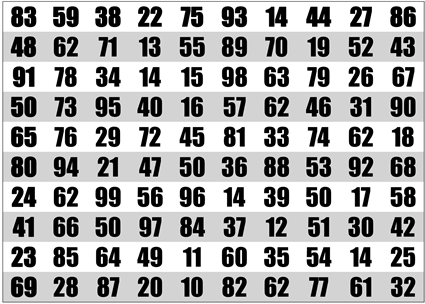


Given figure is the set of 100 numerals in which wanted numerals are marked by red circles. Here numerals 14, 50 and 62 are repeated 4, 4 and 5 times. So totally there are 13 wanted numerals.


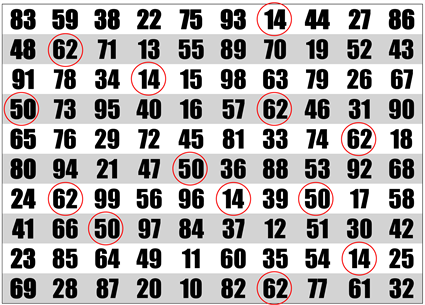


**Typo Revealing (TR) Test**

Given figure is the standard passage used in the TR test and provided to the students to read carefully at a glance.


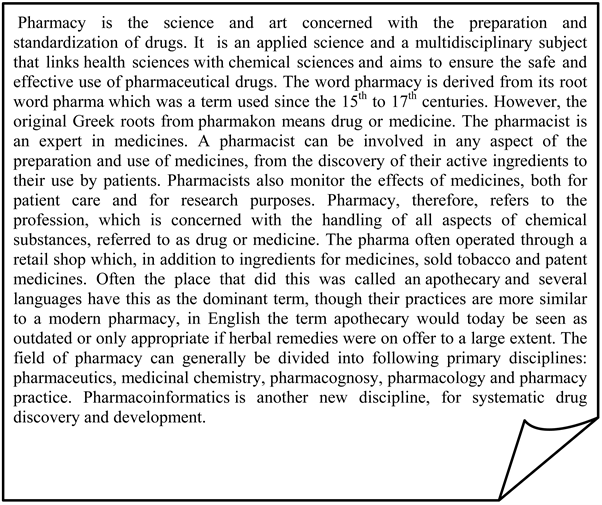


Given figure is the passage with typological mistakes is marked by red circles. This type of mistake may addition of any letter/punctuation (i.e., neuroon instead of neuron; bra!in instead of brain), deletion of any letter/punctuation (i.e., nerv instead of nerve; Alzheimers disease instead of Alzheimer’s disease), substitution of any letter/punctuation (i.e., antiblotics instead of antibiotics; N_acetyl-p-aminophenol instead of N-acetyl-p-aminophenol), inversion of any letter (i.e., sceince instead of science), providing capital letter instead of small letter etc. So totally there are 15 typological mistakes.


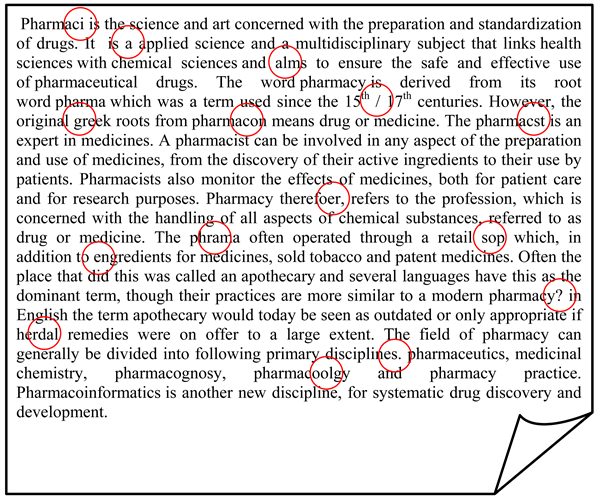

Supplement: Supplementary file 1 [file Data_Sheet_1.doc]
